# Direct-tuning methods for semiconductor metamaterials

**DOI:** 10.1038/s41598-019-54066-5

**Published:** 2019-11-26

**Authors:** Li Min, Wenjin Wang, Lirong Huang, Yonghong Ling, Tongjun Liu, Jing Liu, Chaoming Luo, Qingdong Zeng

**Affiliations:** 1 Department of Physics and Electronics, Hunan Institute of Science and Technology, Yueyang, 414000 P.R. China; 2 Wuhan National Laboratory for Optoelectronics (WNLO), Huazhong University of Science and Technology, Wuhan, 430074 P.R. China; 3 College of Information and Communication Engineering, Hunan Institute of Science and Technology, Yueyang, 414000 P.R. China; 4 School of Physics and Electronic-information Engineering, Hubei Engineering University, Xiaogan, 432000 P.R. China

**Keywords:** Metamaterials, Nanophotonics and plasmonics

## Abstract

Among various tunable optical devices, tunable metamaterials have exhibited their excellent ability to dynamically manipulate lights in an efficient manner. However, for unchangeable optical properties of metals, electromagnetic resonances of popular metallic metamaterials are usually tuned indirectly by varying the properties or structures of substrates around the resonant unit cells, and the tuning of metallic metamaterials has significantly low efficiency. In this paper, a direct-tuning method for semiconductor metamaterials is proposed. The resonance strength and resonance frequencies of the metamaterials can be significantly tuned by controlling free carriers’ distributions in unit cells under an applied voltage. This direct-tuning method has been verified in both two-dimensional and three-dimensional semiconductor metamaterials. In principle, the method allows for simplifying the structure of tunable metamaterials and opens the path to applications in ultrathin, linearly-tunable, and on-chip integrated optical components (e.g., tunable ultrathin lenses, nanoscale spatial light modulators and optical cavities with resonance modes switchable).

## Introduction

Numerous exotic phenomena in metamaterials (e.g., invisible cloak^1^, negative refraction^2^, abnormal reflection^3^) have enabled a considerable number of appealing applications, such as ultra-compact wave plates^4^, broadband absorbers^5^ and optical circuit boards^6^. Moreover, the electromagnetic properties of metamaterials can be freely tuned by lights^7–9^, electrics^10–13^, magnetics^14–17^ and temperatures^18–20^ changing material composition or shapes of metamaterial unit cells. It is known that these optical properties are largely determined by the collective resonance movement of free electrons in metamaterial unit cells. For example, electrons’ effective path length (EPL) determines the resonance wavelength, and the number of free electrons participating in resonance motions regulates the resonance strength^21^.

In fact, most of the reported tunable metamaterials, made of noble metals, are not likely to be tuned directly because free electrons in metallic unit cells are uncontrollable. Existing studies have focused on varying substrates’ properties or structures (e.g., refractive index^22^, GaAs’s depletion width^23^, graphene’s optical constants^24^, ITO’s permittivity^25^, PDMS’s shape^26^ and vanadium dioxide’s conductivity^27^) to tune the metamaterial response. Obviously, compared with direct metamaterial tuning, these indirect tuning methods may not excite the potential tunable ability that metamaterials deserved. On the basis of the control of free electrons in unit cells of metamaterials, direct-tuning method can lead to a sufficiently large free electron distribution and density change in a material (e.g., heavily doped semiconductor or conducting oxide), thus resulting in a large variation for resonance frequency and resonance strength of metamaterials. Predictably, directly tunable metamaterial that will be a distinctly attractive modulation approach because it not only combines advantages of individual modulation of metamaterial elements but also leads to extremely lower power dissipation.

Thus far, however, no comprehensive research has been conducted on direct tuning methods for metamaterials from directly tunable elements. Directly tunable metamaterials require that the electromagnetic property of metamaterial component is variable, and there are many similar materials. For instance, doped semiconductors (e.g., GaAs, InSb, InAs, ZnO) and conducting oxide (e.g., ITO^25^) are typical materials whos free electrons (free carriers) are easy to tune by electric fields, pumping lights or temperature fields, and which can also be used to construct metamaterials^28^. Here, semiconductors (GaAs) were used to construct a two-dimensional (2D) and three-dimensional (3D) semiconductor metamaterials (SMs) to demonstrate a directly tunable metamaterials that allows dynamic electric control. In this work, unit cells of SMs are made of n-doped and p-doped semiconductors to keep the depletion region from disappearing to achieve equilibrium, and substrates are intrinsic GaAs.

## Results

### Directly tunable two-dimensional SMs

**Figure 1:**
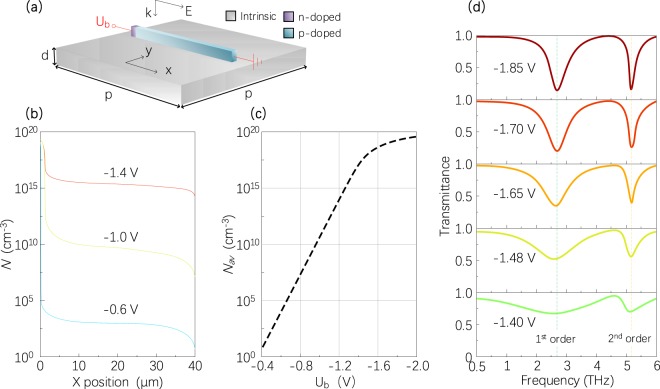
(**a**) Schematic diagram of the unit cell (strip) of 2D SMs on the substrate (intrinsic GaAs) with d = 8 μm and p = 60 μm. The doped-GaAs strips have the length of 40 μm, the width of 4 μm and the thickness of 5 μm. A plane wave is normally incident on the SM, with the electric field along the x direction. (**b**) Spatial distribution of the carrier concentration *N* along the x direction in the strip for different applied voltages. (**c**) Average carrier concentration *N*_*av*_ as a function of applied bias for the exploited voltage between −0.4 V and −2V. (**d**) Transmittance spectra change from the 2D SM for different applied voltages. The green and yellow dash lines represent the positions of transmittance dips corresponding to the first order (1^st^ order) and the second order (2^nd^ order) electric resonances, respectively.

Two-dimensional metamaterials (also known as planar metamaterials), as a type of most popular metamaterials, are usually made of noble metals. Here, a 2D SM is presented, the unit cell of which is depicted in Fig. 1(a). A strip consists of two regions with different types of doping: an n-type region dominated by electrons, and a p-type region dominated by holes. Along the x direction, the n-doped and p-doped regions in the strip have the length of 2 μm and 38 μm and the carrier concentration of 4.0 × 10^−19^ cm^−3^ and 3.8 × 10^−19^cm^−3^, respectively. Unlike previous electrically tunable metamaterials that apply the voltages between the unit cell and the bottom substrate, the voltage bias in this work is employed on unit cells of directly tunable metamaterials. The anode contact is adjacent to the p-type region, and the cathode links to the n-type region. The voltage *U*_*b*_ between the n-doped GaAs and the p-doped GaAs is applied, leading to the formation of free electrons accumulation in the strip. For instance, as the applied voltage *U*_*b*_ increases, the depletion region narrows slowly, and increasing free carriers will gradually fill the entire p-doped region, as shown in Fig. 1(b). The average carrier concentration of *N*_*av*_ in the strip increases with the applied *U*_*b*_, as shown in Fig. 1(c).

Through finite element electromagnetic simulation, the transmittance modulation of the periodically patterned antenna structure was simulated under normal incidence illumination with a transverse electric (TE) polarization (E-field along the stripes, Fig. 1(a)). As mentioned above, when the electrical bias applied on the strip is sufficiently large, free electrons will distribute widely. These free electrons will excite the first and the second order electromagnetic resonances, as shown in Fig. 1(d). In our simulations, it is assumed that when applied voltage *U*_*b*_ is above −0.4 V, free electrons will almost evenly distribute in the strip. As a result, with the increase in gate bias, the EPL of free electrons will remain unchanged, and increasing free electrons will participate in the collective resonance motion when EM waves on the metamaterial. Figure 1(d) suggests that with the rise in the applied voltage, electric resonances (e.g., the 1^st^ order and 2^nd^ order resonances) nearly do not shift to other frequencies; they will be enhanced. It also has been verified with FDTD simulations (see Supplementary Fig. S1). Results indicate that a ∼42% change in the transmittance is achieved by changing the applied voltage bias from *U*_b_ = −1.4 V to *U*_b_ = −2 V at the frequency of 2.7 THz. Besides, it is noteworthy that this linear enhancement phenomenon (i.e., increasing resonance strength without varying the resonance frequencies) can be hardly found in other reported tunable metallic metamaterials and will be very suitable for linearly tunable optical components (e.g., phase retarders and linear modulators).

**Figure 2:**
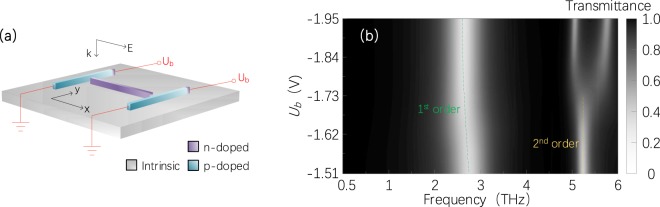
(**a**) Schematic diagram of 2D EIT SMs’ unit cell consisting of three strips on the substrate (intrinsic GaAs). The geometrical parameters of the three strips are the same as the strip shown in Fig. 1(a). A plane wave is normally incident on the SM, with the electric field along the x direction. (**b**) Transmittance spectra change from the 2D EIT SM for different applied voltages *U*_*b*_. The green and yellow dash lines represent the positions of transmittance dips corresponding to the 1^st^ order and 2^nd^ order electric resonances, respectively.

The EPL of free electrons in unit cells can also vary with near-field effects. It is known that the effect of near-field interactions between metallic elements in a metamaterial unit cell on the EPL cannot be neglected, especially when two subwavelength structures are very close, which can excite electromagnetically induced transparency (EIT)^29,30^. In transparent metallic metamaterials, the EIT is usually tuned by varying the relative position of elements in unit cells^31^, adjusting the incident angle or polarization state of light^32,33^, or altering the equivalent structure of elements^30,34^. Here, a tunable transparent SM that enables dynamic electrical control of the EIT without changing the shape or position of elements in unit cells is numerically demonstrated. The unit cell of the transparent SM consists of a strip (made of n-doped GaAs, carrier concentration of ~5 × 10^19^ cm^−3^) and a couple of parallel strips (both the same as that in Fig. 1(a)), as shown in Fig. 2(a).

The strip parallel to the x direction acts as a resonance unit cell to generate the 1^st^ order and 2^nd^ order electric resonances, in which the distribution and carrier concentration of free electrons will be affected by the near field of the other two parallel strips. According to Refs. ^31,35^, under a normal incidence, the fundamental (i.e., the 1^st^ order) resonance in symmetrical-structure unit cells will not be affected seriously by the near field. In this work, the 1^st^ order resonance frequency of the SMs only makes a slight shift (~0.15 THz) when the applied voltage *U*_b_ rises from −1.51 V to −1.95 V, as shown with the green dash line in Fig. 2(b).

For the 2^nd^ order resonance, when the applied voltage *U*_*b*_ = 0, the two strips will have no free electrons and will not couple the incident light to form a strong near field, such that the resonance will not be affected. With the applied voltage increasing from 0 to −1.73 V, the coupled electric field in the two parallel strips increases in magnitude, which gradually changes the spatial distribution of free electrons in the strip, and the 2^nd^ order resonance strength decreases slowly at the resonance frequency of 5.2 THz, as illustrated in Fig. 2(b). When the applied voltage >1.73 V, the transparency window and EIT peak (between two resonance dips around the frequency of 5.2 THz) gradually appear. Under the applied voltage of −1.95 V, the original 2^nd^ order resonance completely disappears, and the EIT peak of 84% is observed in the transmittance spectrum, thus achieving an on-to-off EIT peak modulation. It has been further confirmed with FDTD simulations (see Supplementary Fig. S2). This physical mechanism of the EIT can be well explained by the analogy between the system and atomic EIT systems^31,34^.

Compared with other metallic metamaterials, field-effect modulation especially for directly tunable SMs may be a distinctly attractive approach for its wide distribution in semiconductor electronics, extremely low power dissipation and simpler modulation process (as mentioned above). It will be very suitable for on-chip integrated modulators or switchers.

### Directly tunable three-dimensional SMs

**Figure 3:**
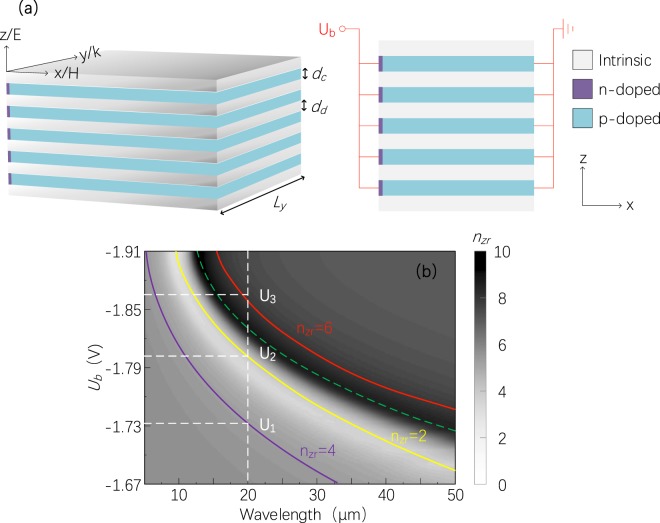
(**a**) Schematic diagram of the tunable 3D SMs with definitions of the geometrical parameters: *L*_*y*_ = 5 μm, *d*_*c*_* = *400 nm and *d*_*d*_ = 400 nm. The 3D SMs consists of 11 alternating layers of intrinsic GaAs and n-p-doped GaAs. A plane wave is incident along the y direction, with the electric field and magnetic field along the z direction and the x direction, respectively. (**b**) Effective refractive index (*n*_*z*_) spectra of 3D SMs in the z direction for different applied voltages *U*_*b*_. in this study, *n*_*z*_ = (*ε*_*z*_)^1/2^, and *ε*_*z*_ is the permittivity in the z direction, expressed as *ε*_*z*_ = (*d*_*c*_* + d*_*d*_)/(*d*_*c*_/*ε*_*c*_ + *d*_*d*_/*ε*_*d*_), where *ε*_*c*_ and *ε*_*d*_ are permittivity of intrinsic and n-p-doped GaAs, respectively. The green dashed line represents the position of the electric resonance. The yellow, purple and red solid lines refer to locations where real parts of the refractive index *n*_*z*_ are 2, 4 and 6, respectively. Dashed lines represent the corresponding values (*U*_2_, *U*_1_ and *U*_3_) of applied voltages when a light with a wavelength of 20 μm leads to the formation of the 1^st^, 2^nd^ and 3^rd^ order resonances in the Fabry-Perot cavity.

Three-dimensional (3D) metamaterials (also known as bulk-like metamaterials) are another type of research interest in recent years^36^. Layered metamaterials, as a type of 3D metamaterials and usually composed of a sequence of dielectric layers (permittivity *ε*_*d*_ > 0) and conductive layers (permittivity *ε*_*c*_ < 0), can be grown with molecular beam epitaxy on lattice-matched substrates^37^. Moving free electrons in conductive layers can create an electric dipole, and it is critical to induce electric resonances in layered 3D metamaterials. Given this principle, noble metals are extensively taken as the conductive layers for its larger number of free electrons to stronger resonances. However, as discussed above, such metamaterials cannot be directly tuned. In fact, few tunable 3D metamaterials have been reported thus far. In this paper, a tunable 3D SM is numerically demonstrated, the resonance of which can be directly controlled with an applied voltage, as shown in Fig. 3(a).

Likewise, as the applied voltage is up-regulated (from 0 to −1.91 V), increasing free electrons diffuse into p-type region in n-p-doped layers. As a result, the increase in free carrier concentration will raise free electrons’ collision rate and give rise to reduction in the EPL of free electrons. In other words, the resonance will shift to shorter wavelengths (shown with the green dashed line in Fig. 3(b)). In the meantime, it will bring about a stronger resonance. For instance, as shown in Fig. 3(b), the variation of refractive index of 3D SMs is as high as 10, which is remarkable when compared with that of naturally occurring substances. Interestingly, it can make the 3D SMs very suitable for realizing a Fabry-Perot cavity with the resonance mode switchable. For instance, at the wavelength of 20 μm, when the applied voltages are *U*_1_ = −1.73 V, *U*_2_ = −1.80 V and *U*_3_ = −1.87 V, and corresponding refractive index of the 3D SMs are *n*_*z*_ = 2, 6 and 4, represented by the yellow, purple and red solid lines, as shown in Fig. 3(b), respectively. Also, according to the resonance condition (2*kL*_*y*_ = 2*m*π, where *k* and *L*_*y*_ are the wave number of cavity and the optical cavity’s length, respectively, and *m* is the order of resonance mode number), this 20 μm-wavelength light can reside in the cavity in different resonance modes, e.g., the 1^st^, 2^nd^ and 3^rd^ order modes (see Supplementary Fig. S3).

It is worthy to note that, doped semiconductors in short-wavelength bands (e.g., near-IR, visible and UV bands) cannot exhibit plasma properties (i.e., *ε*_*c*_ > 0), the proposed cavity will be not available in these bands. Nevertheless, we can choose a low effective-mass and high-mobility semiconductor (e.g., indium tin oxide, zinc oxide doped with aluminum or gallium^38^) or add metallic layers in the layered SMs to alleviate this problem. Compared with previous generations of tunable metamaterials with complex structures (e.g., split-ring resonators, fishnet structure and nanoparticles), such layered SMs are easy to achieve with lithography-free thin-film deposition, which can significantly simplify the process of fabrication^36^.

## Discussion

In conclusion, a direct-tuning method for tunable semiconductor metamaterials (SMs) is proposed, and its modulation mechanisms are reported by studying the distribution regularity of free electrons in unit cells. Results show that both the resonance frequency (or wavelength) and resonance strength of 2D and 3D SMs can be directly tuned with an applied voltage. These directly tunable SMs are promising candidates as a novel of ultra-compact and high-performance tunable optical elements and can find new possibilities for realizing integration with electronics. With the suggested methodology, other stimuli (e.g., pump lights and temperature fields) can also be used to directly tune semiconductor metamaterials. It may open a new roadmap for tunable metamaterial-based components and devices.

## Methods

In the study, the numerical simulations were performed by commercial software COMSOL Multiphysics. In the simulation of free carrier distributions (e.g., spatial distribution and concentrations) in unit cells of SMs, some key physical parameters of GaAs (e.g., relative permittivity, electron mobility, hole mobility and bandgap voltage) were set as 12.9, 8500 cm^2^/(V·s), 400 cm^2^/(V·s) and 1.424 V, respectively, in the Semiconductor Module of COMSOL soft. In the calculation of the permittivity of doped GaAs and the effective refractive index of SMs, relevant theories and equations in Refs. ^39–41^ are adopted in this study. In the simulation of transmittance spectra of SMs, periodic boundary condition was set in y direction, and perfectly matched layer condition was applied in the x direction in the RF Module of COMSOL softs.

## Supplementary information

Supplementary Information
