# Supplementary material for: Direct-tuning methods for semiconductor metamaterials
Source: Sci Rep. 2019 Nov 26;9:17622. doi: 10.1038/s41598-019-54066-5 (PMC6879632; doi:10.1038/s41598-019-54066-5)
Supplement: Supplementary file 1 — Supplementary Information [file 41598_2019_54066_MOESM1_ESM.pdf]

Supplementary Information

Direct-tuning methods for semiconductor metamaterials

Li Min*, Wenjin Wang, Lirong Huang, Yonghong Ling, Tongjun Liu, Jing Liu, Chaoming Luo, Qingdong Zeng.

*To whom correspondence should be addressed; email: min@hnist.edu.cn

To confirm the results of the main text, we have performed additional simulations for doped semiconductor metamaterials (SMs) with Lumerical FDTD Solutions, a commercially available finite-difference time-domain (FDTD) simulation software package. Electric fields are detected within the frequency profile monitors. To model the materials in the simulations, we used proper formulations as mentioned in the main text and in Supplementary Information.

Supplementary simulations for tunable 2D SMs

As we know, both permittivity and conductivity of doped semiconductors are closely related to their free carrier concentrations [S1-S5]. As for doped GaAs, the relationship between permittivity and free carrier density can be described with Supplementary Fig. S1 (a), and the relationship between conductivity free carrier density can be depicted with Supplementary Fig. S1 (b). Such that, we can perform FDTD simulations of the tunability of doped SMs. Here, we simulate transmittance spectra of 2D SMs with the FDTD soft for different free carrier densities, the unit cell of which is an I-shaped doped metamaterial same with that in Fig.1(a) of the main text, and show the results in Supplementary Fig .S1(c).

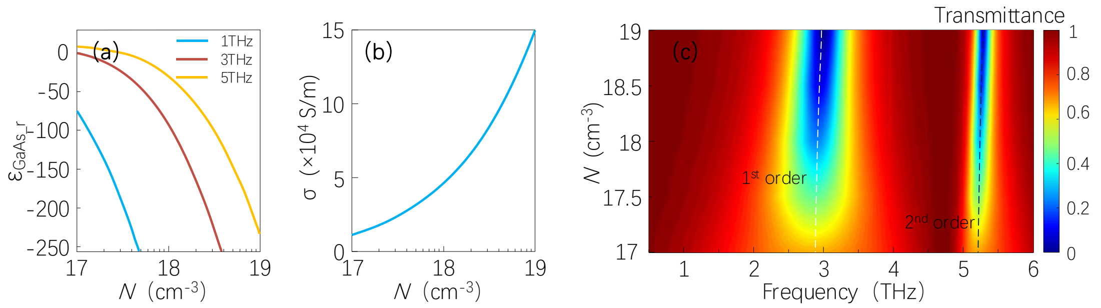

Supplementary Fig. S1 (a) Real parts ε_{GaAs_r} of permittivity and (b) conductivity σ of the doped GaAs change with free carrier densities *N*. (c) Transmittance spectra of I-shaped doped metamaterials vary with free carrier densities *N* of doped GaAs. The black dash line and white dash line represent the positions of transmittance dips corresponding to the first order (1st order) and the second order (2nd order) electric resonances, respectively.

We can see that, the real parts $|\varepsilon_{GaAs\_r}|$ of the relative permittivity (see Supplementary Fig. S1 (a)) and conductivity σ (see Supplementary Fig. S1 (b)) of doped GaAs increase with the free carrier density in doped GaAs. Such that, both the first order (1st order) and second order (2nd order) electric resonances become stronger and stronger, as shown in Supplementary Fig. S1 (c). As discussed in the main text, the applied voltage will give rise to the increase in the average free carrier density N_{av} (see Fig. 1(c) of the main text). Consequently, the results (see Supplementary Fig. S1 (c)) are consistent with COMSOL simulation results (see Fig. 1(d) of the main text) in main text.

Similarly, we performed FDTD simulations of the tunability of the electromagnetically induced transparency (EIT) of 2D SMs. Both the structural parameters of the unit cell of transparent SMs and the polarization state of incident waves are same with that in Fig.2(a) of the main text. Simulated transmittance spectra of the transparent doped metamaterials for different free carrier densities of doped GaAs are shown in Supplementary Fig .S2.

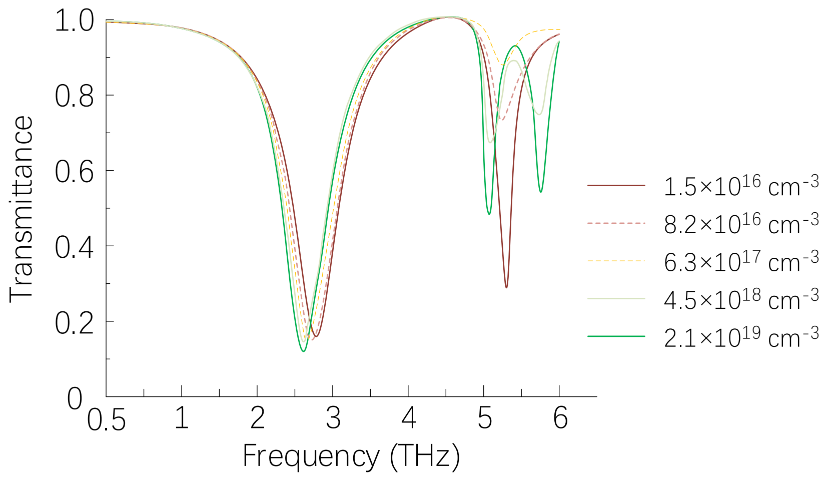

Supplementary Fig. S2 Transmittance spectra of EIT metamaterials vary with free carrier densities N in doped GaAs.

We can see from Supplementary Fig. S2, when the free carrier density in the two parallel strips increases from $1.5\times10^{16}cm^{-3}$ to $2.1\times10^{19}cm^{-3}$, the electric resonance at the frequency of ~ 5.2THz becomes weaker and gradually disappears, and yields two new electric resonances at frequencies of ~ 5.1THz and ~ 5.8THz, respectively, namely, an EIT occurs. Furthermore, as the free carrier density increases, the EIT is more and more obvious. The result of tunability of 2D SMs using FDTD softs coincides with that (see Fig.2(b) of the main text) using COMSOL softs in this paper.

Supplementary simulations for tunable 3D SMs

Here we provide additional simulations for directly tunable three-dimensional (3D) SMs (layered metamaterials) with FDTD softs. Both the structural parameters of the unit cell of the 3D SMs and the polarization state of incident waves are same with that in Fig.3(a) of the main text. Multilayer metamaterials can be treated as a homogeneous effective medium and the anisotropic refractive index tensor component can be determined from the Maxwell-Garnet theory [S6, S7]. The effective refractive index along the direction normal to layers of the 3D

layered SMs (see Fig.3(a) of the main text) can be expressed with,

$$n_z = \sqrt{\frac{d_c + d_d}{d_c / \varepsilon_c + d_d / \varepsilon_d}} \quad \text{(S1)}$$

At a wavelength of 20μm, when the free carrier densities of doped GaAs are $6.3\times10^{-18}cm^{-3}$, $1.3\times10^{-19}cm^{-3}$ and $2\times10^{-19}cm^{-3}$, the real parts n_{zr} of effective index of the 3D layered SMs can be calculated based on the relationship between permittivity and free carrier density discussed above and Eq. S1, and are 2, 4 and 6, respectively, and the corresponding electric field distribution in the 3D layered SMs has been simulated, as shown with Supplementary Fig. S3.

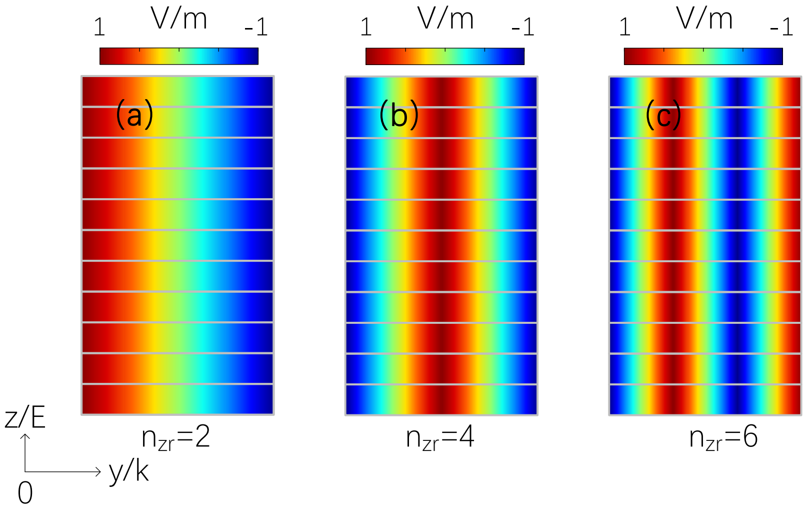

Supplementary Fig. S3 (a), (b) and (c) show electric field distributions of YOZ cross-section of 3D layered SMs when real parts n_{zr} of effective refractive index n_z are 2, 4 and 6, respectively. Here, the plane wave with a wavelength of 20μm is incident along the y direction, with the electric field and magnetic field along the z direction and the x direction, respectively.

As we can see from Supplementary Fig. S3, the 3D layered SMs could be considered as a tunable Fabry-Perot cavity, in which this 20μm-wavelength light can reside, and form the 1st, 2nd and 3rd order Fabry resonances when n_{zr} equals to 2, 4 and 6, respectively. This result is consistent with that expected in the main text.

Supplementary References

[S1] M. Cardona, "Electron effective masses of InAs and GaAs as a function of temperature and doping," Physical Review **121**(3), 752 (1961).

[S2] D. Shrekenhamer, S. Rout, A. C. Strikwerda, C. Bingham, R. D. Averitt, S. Sonkusale, and W. J. Padilla, "High speed terahertz modulation from metamaterials with embedded high electron mobility transistors," Optics express **19**(10), 9968-9975 (2011).

[S3] W. Cai, and V. M. Shalaev, *Optical metamaterials* (Springer, 2010).

[S] M. Wang, R. Vajtai, P. M. Ajayan, and J. Kono, "Electrically tunable hot-silicon terahertz attenuator," J Applied Physics Letters **105**(14), 141110 (2014).

[S4] I. Gil, J. Bonache, J. Garcia-Garcia, F. Martin, and techniques, "Tunable metamaterial

transmission lines based on varactor-loaded split-ring resonators," IEEE transactions on Microwave theory **54**(6), 2665-2674 (2006).
[S5] X. Yang, Y. Jie, J. Rho, X. Yin, and Z. Xiang, "Experimental realization of three-dimensional indefinite cavities at the nanoscale with anomalous scaling laws," Nature Photonics **6**(7), 450-454 (2012).
[S6] A. H. Sihvola, *Electromagnetic Mixing Formulas and Applications* (The Institution of Electrical Engineers, London, 1999).
